# A Bio-Inspired Artificial Nerve Simulator for Ex Vivo Validation of Implantable Neural Interfaces Equipped with Plug Electrodes

**DOI:** 10.3390/bioengineering12121366

**Published:** 2025-12-16

**Authors:** Daniel Mihai Teleanu, Octavian Narcis Ionescu, Carmen Aura Moldovan, Marian Ion, Adrian Tulbure, Eduard Franti, David Catalin Dragomir, Silviu Dinulescu, Bianca Mihaela Boga, Ana Maria Oproiu, Ancuta Diana-Larisa, Vaduva Mariana, Coman Cristin, Carmen Mihailescu, Mihaela Savin, Gabriela Ionescu, Monica Dascalu, Mark Edward Pogarasteanu, Marius Moga, Mirela Petruta Suchea

**Affiliations:** 1 University of Medicine and Pharmacy “Carol Davila”, 050474 Bucharest, Romania; daniel.teleanu@umfcd.ro (D.M.T.); bianca-mihaela.boga@drd.umfcd.ro (B.M.B.); anamaria.oproiu@umfcd.ro (A.M.O.); mark.pogarasteanu@umfcd.ro (M.E.P.); marius.moga@umfcd.ro (M.M.); 2 IMT Bucharest, 077190 Bucharest, Romania; carmen.moldovan@imt.ro (C.A.M.); marian.ion@imt.ro (M.I.); eduard.franti@imt.ro (E.F.); david.dragomir@imt.ro (D.C.D.); silviu.dinulescu@imt.ro (S.D.); carmen.mihailescu@imt.ro (C.M.); mihaela.savin@imt.ro (M.S.); 3 Petroleum and Gas University from Ploiesti, 100680 Ploiesti, Romania; gabriela.ionescu@upg-ploiesti.ro; 4 Faculty of Electrical Engineering, Universitatea “1 Decembrie 1918”, 510009 Alba Iulia, Romania; aditulbure@uab.ro; 5 Preclinical Testing Unit, The Technological Research and Development Center, Cantacuzino National Military-Medical Institute for Research and Development, 050096 Bucharest, Romania; ancuta.diana@cantacuzino.ro; 6 Experimental Medicine and Translational Research Platform, The Technological Research and Development Center, Cantacuzino National Military-Medical Institute for Research and Development, 050096 Bucharest, Romania; vaduva.mariana@cantacuzino.ro (V.M.); coman.cristin@cantacuzino.ro (C.C.); 7 Faculty of Veterinary Medicine, University of Agronomic Sciences and Veterinary Medicine, 011464 Bucharest, Romania; 8 National University of Science and Technology Politehnica, 061085 Bucharest, Romania; monica.dascalu@upb.ro; 9 Center of Materials Technology and Photonics, School of Engineering, and Centre for Research and Innovation (PEK), Hellenic Mediterranean University (HMU), 71410 Heraklion, Greece

**Keywords:** bio-inspired artificial nerve simulator, neural interface, ex vivo test platform

## Abstract

The development of implantable neural interfaces is essential for enabling bidirectional communication between the nervous system and prosthetic devices, yet their evaluation still relies primarily on in vivo models which are costly, variable, and ethically constrained. Here, we report a bio-inspired artificial nerve simulator engineered as a reproducible ex vivo platform for pre-implantation testing of plug-type electrodes. The simulator is fabricated from a conductive hydrogel composite based on reduced graphene oxide (rGO), polyaniline (PANI), agarose, sucrose, and sodium chloride, with embedded conductive channels that replicate the fascicular organization and conductivity of peripheral nerves. The resulting construct exhibits impedance values of ~2.4–2.9 kΩ between electrode needles at 1 kHz, closely matching in vivo measurements (~2 kΩ) obtained in *Sus scrofa domesticus* nerve tissue. Its structural and electrical fidelity enables systematic evaluation of electrode–nerve contact properties, signal transmission, and insertion behavior under controlled conditions, while reducing reliance on animal experiments. This bio-inspired simulator offers a scalable and physiologically relevant testbed that bridges materials engineering and translational neuroprosthetics, accelerating the development of next-generation implantable neural interfaces.

## 1. Introduction

Implantable neuroprosthetic systems that establish bidirectional communication with the human nervous system remain one of the central challenges in biomedical engineering. By acquiring neural signals that encode motor intentions and by delivering sensory feedback from prosthetic devices, such systems aim to restore lost function and improve the quality of life for patients with limb loss or paralysis. However, the road from conceptual design to clinical translation is still constrained by a fundamental bottleneck: the lack of reliable, reproducible, and physiologically relevant platforms for testing electrode–nerve interactions before in vivo experimentation and implantation.

Traditionally, the validation of implantable neural interfaces has relied heavily on animal models. Although such models provide invaluable physiological insight, they are associated with ethical concerns, high costs, and significant biological variability, all of which complicate systematic optimization. Many early-stage engineering questions—such as tuning impedance characteristics, optimizing electrode geometry, or calibrating insertion forces—do not necessarily require living tissue but rather a controlled, biomimetic environment that captures the essential structural and electrical features of peripheral nerves. This need has driven the development of synthetic nerve phantoms and tissue simulators for in vitro testing of electrodes, stimulators, and recording systems.

In parallel, recent advances in artificial nerve systems and nerve-on-a-chip models underline the growing interest in engineered platforms for studying neural interfaces. Piezoionic and conductive hydrogel-based artificial nerves have been proposed for neuromodulation and signal processing, but these systems are typically designed for sensing or therapeutic stimulation rather than for standardized ex vivo electrode testing [1,2,3]. Likewise, nerve-on-a-chip platforms and biomimicking microfibers provide powerful microphysiological models for nerve regeneration and drug testing, yet they focus on cellular-scale biology rather than macroscopic impedance-matched phantoms [4,5,6]. Recent soft robotic neural cuffs are often evaluated on simplified nerve phantoms before animal implantation, further highlighting the need for well-defined, physiologically relevant artificial nerve simulators that bridge benchtop characterization and in vivo validation [2].

Existing artificial nerve models, however, still present important limitations. Many are fabricated from inert silicone or polymer matrices that approximate bulk mechanical properties but do not reproduce ionic conduction or physiological impedance. Others incorporate conductive channels but lack realistic fascicular organization or long-term hydration stability. As a result, current simulators rarely capture the combined structural and functional complexity of peripheral nerves, which limits their utility for advanced neuroprosthetic development. Furthermore, standardized platforms for evaluating electrode insertion behavior, contact impedance, and signal transmission fidelity remain scarce and slow the translation of new electrode designs from the laboratory to the clinic. Recent reviews on peripheral nerve interfaces and neuroprosthetic systems similarly emphasize the lack of standardized, physiologically relevant platforms for pre-clinical testing of electrode–nerve interactions [7,8].

In this context, we present the design, fabrication, and validation of a bio-inspired artificial nerve simulator that addresses these critical challenges. Developed within the framework of the NerveRepack project—a European initiative focused on creating intelligent neural systems for bidirectional connection with prostheses—the simulator is specifically engineered to reproduce both the fascicular architecture and the electrical properties of human peripheral nerves. Its core consists of a conductive hydrogel composite based on reduced graphene oxide (rGO), polyaniline (PANI), agarose, sucrose, and sodium chloride, which together provide ionic conductivity, stable impedance, and bio-relevant hydration dynamics. Embedded within this matrix are fascicle-mimicking conductive channels that enable controlled evaluation of plug-type electrodes—a promising alternative to traditional cuff electrodes for neural interfacing.

This article details the complete development of the artificial nerve simulator, including material selection, fabrication workflow, structural and electrical characterization, and validation using experimental electrode assemblies. We demonstrate that the simulator exhibits impedance values (~2 kΩ at 1 kHz) closely matching in vivo peripheral nerve measurements and supports stable, reproducible signal transmission. Finally, we discuss its potential applications not only as a pre-implantation testing platform for implantable modules but also as a versatile tool for iterative electrode optimization, surgical training, and standardized benchmarking of neuroprosthetic devices. By bridging materials innovation and engineering design, this work establishes a foundational step toward safer, faster, and more efficient development of next-generation neural interfaces.

### State of the Art in Artificial Nerve Models and Neural Interface Testing

The development and validation of implantable neural interfaces have long depended on in vivo animal models, but those bring challenges of reproducibility, cost, ethical constraints, and biological variability. To mitigate these issues, researchers have developed artificial nerve phantoms or simulators that mimic certain mechanical or electrical properties of neural tissue. Many existing models are constructed from inert elastomers or silicone matrices (e.g., PDMS or silicone phantoms) and can include conductive fillers or channels to emulate basic conduction, but they often lack ionic conduction, realistic fascicular structures, or stable long-term hydration. One commonly adopted phantom strategy uses PDMS-based tissue-mimicking phantoms for optical or mechanical modeling, for example, in optical scattering and imaging studies [9].

In parallel, the field of conductive polymers and hydrogel composites has advanced significantly in interfacing electronics with biological tissue. For instance, Lunghi et al. demonstrated PEDOT:PSS micropillars on PDMS substrates to create flexible neural interfaces with improved conformity and enhanced electrical performance [10]. Similarly, other works report stretchable PEDOT:PSS/PDMS devices for simultaneous recording and deformation sensing [11]. These advances underline that soft, conductive, and hydrated materials are increasingly favored to reduce mechanical mismatch and stabilize electrode–tissue interfaces.

Regarding electrode technologies themselves, the community has advanced from extraneural cuff electrodes to deeper intraneural designs (e.g., longitudinal intrafascicular electrodes, transverse multichannel electrodes), aiming for higher selectivity and lower stimulation thresholds. A more recent concept is the plug-type electrode, which penetrates directly into the nerve fascicle, promising more precise interfacing. In our prior work, “*System of Implantable Electrodes for Neural Signal Acquisition and Stimulation for Wirelessly Connected Forearm Prosthesis*”, we presented our newly developed plug microelectrodes and demonstrated their function [12].

Despite these advances, existing simulators rarely simultaneously replicate *fascicular architecture*, *ionic conduction*, and *impedance behavior* in a stable, repeatable format. This gap limits robust ex vivo evaluation of plug electrode designs. The artificial nerve simulator presented here bridges that gap by combining a conductive hydrogel composite (rGO–PANI–agarose–sucrose–NaCl) with embedded fascicle-mimicking conductive channels, yielding impedance (~2.4–2.9 kΩ between electrodes at 1 kHz) closely aligned with in vivo data and enabling rigorous testing of neural interfaces in a controlled environment.

Early approaches focused primarily on mimicking gross nerve morphology and mechanical properties. Silicone, PDMS, thermoplastic polyurethane (TPU), and epoxy-based structures have been used to create cylindrical substrates with diameters in the range of 2–6 mm, approximating the dimensions of human peripheral nerves and supporting the integration of embedded conductors or electrode channels [13,14,15,16,17,18,19,20]. Table 1 of this work summarizes representative examples, including silicone-based conduits with 10-channel molded cores [16,17] and 3D-printed nanostructured sheaths [20]. These designs reproduce surface texture, elasticity (≈10–100 kPa), and macrostructure, but they often lack **ionic conduction** or fail to capture the fascicular organization of real nerves.

Efforts to incorporate electrical functionality have used materials such as PEDOT:PSS, carbon nanotube pastes, silver–epoxy composites, and graphene inks, each offering a balance of conductivity and processability [21,22,23,24,25,26]. For instance, Koutsouras et al., (2019) developed a multi-channel PDMS elastomer filled with PEDOT:PSS hydrogel, achieving conductivities in the range of 1–10 S/m and enabling selective stimulation and impedance profiling [21]. Similarly, Vlasceanu et al., (2019) demonstrated graphene inks printed into TPU matrices to produce flexible conductive paths with crosstalk and attenuation characteristics relevant to in vitro testing [26]. Silver–epoxy composites with conductivities exceeding 10^5^ S/m [25] and carbon-based electrodes [23,24] have also been investigated for simulating axonal conduction paths. A selection of representative material properties is summarized in Table 2.

Advances in fabrication techniques—including soft lithography [27], extrusion-based 3D printing [28], and laser micromachining [29]—have enabled nerve-mimicking constructs with microscale resolution and complex internal channel architectures. For example, Lienemann et al. fabricated gold-nanowire embedded epoxy conduits using direct-write 3D printing and demonstrated consistent signal propagation and impedance spectra under square-wave stimulation [30]. Mishra et al. printed layered graphene–polymer composites that model signal delay and attenuation phenomena observed in demyelinated fibres [31]. Bianchi et al. employed nanotextured PDMS surfaces to study how perineurial topography influences stimulation thresholds [32]. Table 3 summarizes the range of techniques and their corresponding functional outputs.

Electrical testing protocols for nerve simulators typically include impedance spectroscopy (EIS), pulse response, and multichannel signal mapping [30,33,34]. Linemann applied EIS across modular PDMS structures with silver conductors, producing benchmark data for commercial cuff electrodes [30]. Xu et al. reported segmental impedance mapping using carbon-based inks of varying resistivity [35]. The diversity of these approaches highlights both the progress made and the limitations that persist; while many existing phantoms can emulate specific aspects of neural tissue, few simultaneously reproduce fascicular organization, physiologically relevant ionic conductivity, and stable long-term electrical properties. Table 4 presents electrical testing protocols for nerve simulators.

In parallel, electrode design has evolved from traditional extraneural cuff electrodes toward more selective intraneural interfaces, such as longitudinal intrafascicular electrodes (LIFE), transverse intrafascicular multichannel electrodes (TIME), and radial structures. A more recent approach—the plug-type electrode—uses fine needle-like conductors inserted directly into nerve fascicles, enabling highly selective stimulation and signal acquisition. Our recent work, *System of Implantable Electrodes for Neural Signal Acquisition and Stimulation for Wirelessly Connected Forearm Prosthesis* demonstrated the functionality of such electrodes in in vivo experiments [12]. Table 5 presents impedance and capacitance of peripheral nerve fascicles (Sus scrofa domesticus, ~50 kg) values, measured at different frequencies and applied voltages using plug-type electrodes. The contact impedance determined from these measurements was approximately 2 kΩ, which serves as a benchmark for the performance evaluation of the artificial nerve simulator.

Despite these advances, the field still lacks standardized ex vivo testing platforms that combine bio-relevant impedance, fascicular topology, and reproducibility. The artificial nerve simulator described in this work addresses this unmet need. It integrates a conductive hydrogel composite (rGO–PANI–agarose–sucrose–NaCl) with fascicle-mimicking conductive channels, yielding impedance values (≈2.4–2.9 kΩ between electrodes at 1 kHz) that closely align with in vivo measurements. This makes it a powerful tool for electrode testing, optimization, and surgical training, bridging the gap between materials development and clinical translation. A comparative overview of recent artificial nerve simulators and related platforms is provided in Table A1 (Appendix A.2), highlighting their materials, fabrication strategies, electrical performance, and applications.

## 2. Materials and Methods

### 2.1. Design Requirements and Functional Objectives

**Figure 1:**
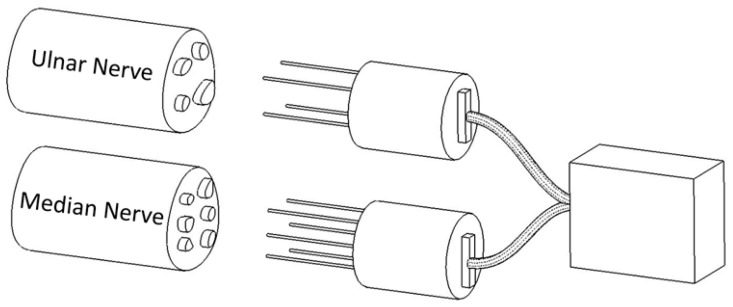
Detailed schematic representation of the plug-type intraneural electrode, designed for direct insertion into peripheral nerve fascicles. This electrode consists of three fine conductive needles configured to achieve selective stimulation and signal acquisition while minimizing mechanical damage to neural tissue. The design enables localized contact with neural fibers, improving selectivity and lowering stimulation thresholds compared to conventional cuff electrodes. Adapted from [12].

While most current neuroprosthetic systems rely on extraneural cuff electrodes, studies have demonstrated that plug-type intraneural electrodes (Figure 1) offer superior selectivity and lower stimulation thresholds due to their direct insertion into nerve fascicles [12]. Their fine geometry allows precise electrode placement while minimizing mechanical damage to neural tissue. This electrode concept forms the basis for the neural interfaces investigated in the NerveRepack project and provides the performance benchmarks used in the present study.

**Figure 2:**
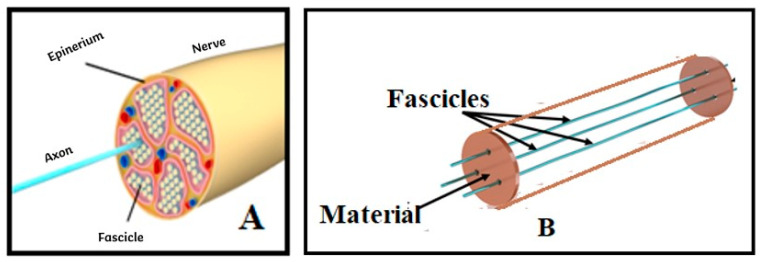
(**A**) Schematic illustration of peripheral nerve fascicular topography, highlighting the epineurium and fascicle distribution. (**B**) Geometry of the developed artificial nerve simulator, in which conductive wires replace the nerve fascicles and the epineurium is mimicked by the conductive hydrogel composite. This biomimetic design reproduces both the structural and electrical characteristics of peripheral nerves.

The geometric design of the artificial nerve simulator (Figure 2B) was based on the fascicular organization of peripheral nerves (Figure 2A). Conductive wires were used to represent fascicles, while the epineurium was replaced by the newly developed hydrogel composite, creating a structure capable of replicating the conductive pathways and impedance behavior of biological tissue.

**Figure 3:**
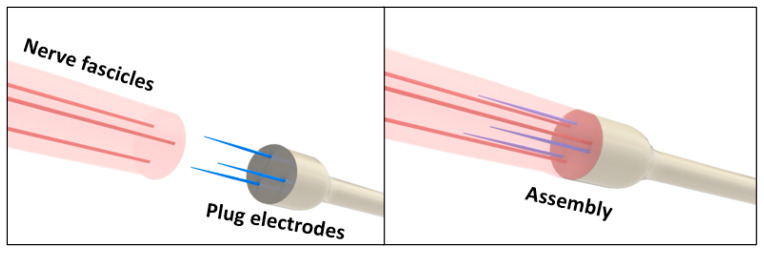
Schematic representation of the configuration of the plug electrode assembly interfaced with a peripheral nerve fascicle. The three-needle geometry enables precise insertion into the fascicular structure, maintaining stable contact and low impedance while minimizing neural damage. This configuration guided the structural design of the artificial nerve simulator.

The artificial nerve simulator was designed to replicate the fascicular geometry observed in in vivo nerve–electrode assemblies (Figure 3). The simulator includes conductive channels mimicking fascicles and allows insertion of plug-type electrodes under controlled conditions, enabling impedance characterization and assessment of electrode contact behavior.

The artificial nerve simulator was designed to provide a physiologically relevant platform for the ex vivo evaluation of implantable neural interfaces, with a focus on plug-type electrodes intended for bidirectional communication between peripheral nerves and prosthetic devices. The design objectives were guided by three primary criteria:

(1) Structural mimicry—replication of the fascicular architecture and cylindrical topology of human peripheral nerves;
(2) Electrical fidelity—reproduction of impedance values and conductive behavior comparable to in vivo peripheral nerve tissue (~2 kΩ at 1 kHz);
(3) Experimental relevance—compatibility with in vitro electrode testing protocols, including impedance mapping and contact characterization.

To achieve these goals, the simulator integrates a conductive hydrogel composite that mimics the ionic and electronic properties of neural tissue with embedded conductive channels that represent nerve fascicles. The resulting construct provides a reproducible test environment for plug-type electrode insertion, signal transmission, and impedance characterization.

### 2.2. Materials and Equipment

The selection of materials was based on the need to ensure biocompatibility, ionic conductivity, and structural stability. Reduced graphene oxide (rGO) and polyaniline (PANI) were chosen as the primary conductive components due to their high electrical conductivity and suitability for biomedical applications. The hydrogel matrix consisted of agarose, sucrose, and sodium chloride (NaCl), which together provided mechanical support, ionic transport pathways, and hydration stability. Ethylene glycol was incorporated to improve the uniformity and stability of the gel network.

Graphene and its derivatives are of particular interest for biomedical devices due to their exceptional conductivity and zero bandgap behavior. However, their poor solubility limits their direct integration into polymeric systems. This limitation was addressed by combining rGO with PANI during a polymerization reaction, which generates functional groups on the graphene surface and improves dispersion within the hydrogel matrix. Additional processing techniques, including sonication and chemical exfoliation, were used to further improve the dispersion and interfacial interaction of rGO within the composite.

### 2.3. Preparation of the Conductive Hydrogel Composite

The conductive hydrogel was prepared by incorporating the conductive polymer PANI and rGO into an agarose-based matrix. Several trials were conducted in order to establish the suitable ratio of rGO:PANI. The main optimization criterion was obtaining a conductivity as close as possible to the nerve. As no multiple use of the same simulator was intended at this point of the study, no mechanical properties and aging studies were performed.

The preparation process consisted of the following steps:

1. A mixture of 1 wt.% PANI powder and rGO (aniline:rGO = 1:2 wt.%—although mechanical tests were not performed, this ratio was observed to produce stable gels without cracking, while higher rGO loading caused brittleness and lower PANI loading reduced conductivity), 2 wt.% sucrose, and 1 wt.% NaCl was dispersed in deionized water and ultrasonicated for 3 min to achieve uniform dispersion.
2. 1 wt.% agarose and 5 wt.% ethylene glycol were then added to the solution.
3. The thermal polymerization of agarose was carried out using microwave heating for two cycles of 20 s at medium power (450 W), ensuring complete dissolution and homogenization.
4. The solution was cooled to approximately 37 °C, after which it was injected into 3D-printed cylindrical molds using a syringe.
5. Three tensioned wires were placed along the length of the molds to simulate nerve fascicles and to carry currents comparable to those present in biological nerves.
6. The molds, with internal diameters of 6 mm and 10 mm, were refrigerated at 4–8 °C for four days to allow full gelation and stabilization.
7. After this period, the screws were removed, and the hydrogel-based artificial nerves were carefully extracted from the molds.

**Figure 4:**
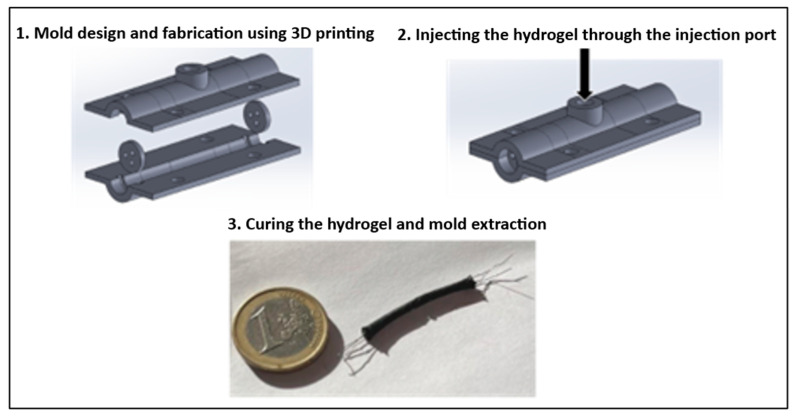
*Fabrication workflow of the artificial nerve simulator*. Schematic illustration of the fabrication process, starting from the preparation of the conductive hydrogel composite (rGO–PANI–agarose–sucrose–NaCl) and continuing with injection into 3D-printed cylindrical molds, placement of tensioned conductive wires to simulate nerve fascicles, and refrigeration for four days at 4–8 °C to achieve full gelation. After demolding, the resulting cylindrical simulators with embedded conductive channels replicate the geometry and ionic conductivity of peripheral nerves.

The overall fabrication workflow is schematically summarized in Figure 4.

### 2.4. Experimental Setup for Electrical Characterization

Two types of artificial nerve simulators were fabricated and tested, differing in diameter (5 mm and 10 mm). The variation in geometry enabled assessment of how structural size influences dehydration kinetics and stabilization of electrical properties. Although smaller (2–3 mm) simulators were also tried, their reduced volume led to excessive dehydration and mechanical fragility, making them unsuitable for reproducible testing. Since the electrical behavior is governed by ionic pathways rather than absolute size, the impedance scaling between 10→5→3.5 mm was linear and consistent (Appendix A.1), supporting extrapolation to physiologically relevant diameters. The larger-diameter structure exhibited faster resistance stabilization, likely due to accelerated dehydration processes and more rapid formation of a stable conductive network within the gel matrix.

Electrical characterization was conducted using impedance spectroscopy at a fixed frequency of 1 kHz and a signal amplitude of 1 Vpp. Measurements were performed in three configurations:

- Between fascicle wires to evaluate intrinsic conductive behavior.
- Between electrode needles to characterize electrode–simulator contact impedance.
- Between electrode needles and fascicle wires to assess signal transmission across the interface.

The measurement setup is shown in Section 3.

## 3. Results and Discussion

### 3.1. Fabricated Nerve Simulators: Geometry and Structural Stability

**Figure 5:**
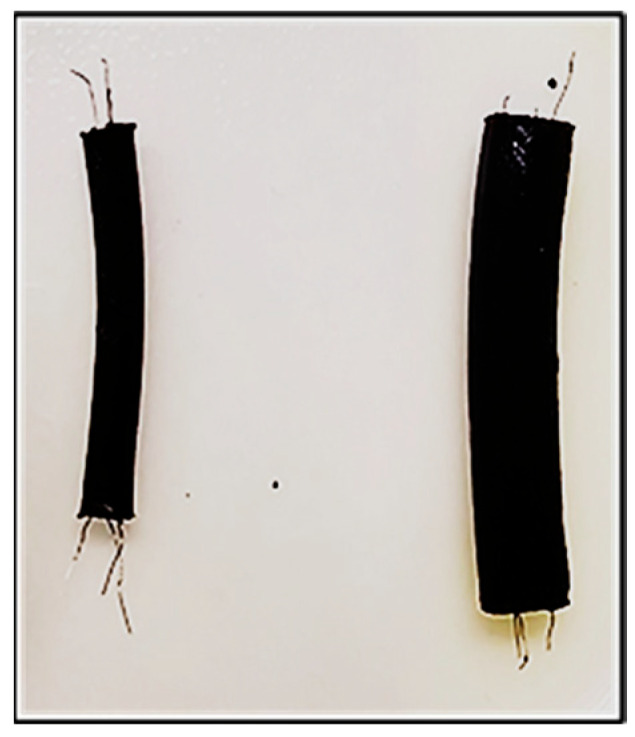
Photographs of the fabricated artificial nerve simulators with diameters of 5 mm and 10 mm.

Two artificial nerve simulators with diameters of 5 mm and 10 mm were fabricated following the procedure described in Section 2 (Figure 5). The cylindrical geometry reproduces the gross morphology of peripheral nerves, and embedded conductive wires represent nerve fascicles. Variations in diameter influence dehydration dynamics and electrical stabilization behavior, with larger simulators showing faster impedance stabilization due to more rapid water loss and conductive network formation. Additional experimental data regarding dehydration and stabilization behavior are provided in Appendix A.1. together with some details of the key fabrication steps to ensure the method can be reliably reproduced.

**Figure 6:**
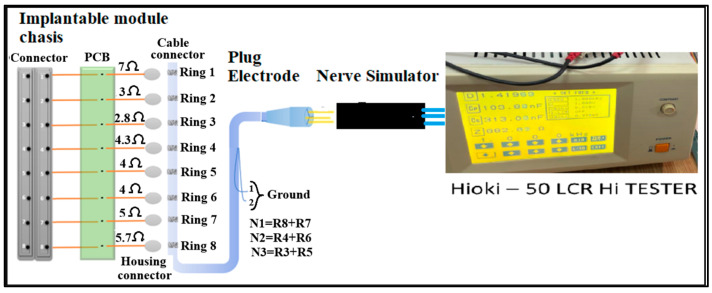
*Experimental setup prepared for* in vitro *electrical testing of the fabricated artificial nerve simulators*. Two simulator samples with diameters of 5 mm and 10 mm were fabricated and mounted for impedance characterization. Variation in simulator diameter influences the dehydration dynamics and stabilization of electrical properties, with larger-diameter samples stabilizing more quickly due to faster water loss and earlier formation of a stable conductive network.

The cylindrical geometry reproduces the macroscopic structure of peripheral nerves, while the embedded conductive wires simulate fascicular pathways within the hydrogel composite. The cylindrical geometry was chosen to replicate the gross morphology of human peripheral nerves, which typically range from 2–6 mm in diameter and contain multiple fascicles organized within an epineurial sheath. The internal conductive channels formed by tensioned wires emulate the fascicular arrangement and provide discrete pathways for electrical conduction, enabling controlled impedance and signal transmission measurements under reproducible conditions. The two geometries exhibited notable differences in their dehydration dynamics. The larger-diameter (10 mm) structures showed faster stabilization of resistance values compared to the smaller-diameter ones. This behavior is attributed to the more rapid water loss in the larger gel volume, which leads to earlier formation of a stable conductive network. In contrast, the smaller-diameter structures underwent slower dehydration, and their electrical properties stabilized over a longer period. These observations underline the influence of geometric parameters on the temporal evolution of the hydrogel’s conductive properties and have practical implications for simulator performance during extended ex vivo testing. Figure 6 presents the experimental setups prepared for in vitro testing.

### 3.2. Impedance Characterization and Electrical Behavior

The electrical behavior of the artificial nerve simulators was evaluated through impedance measurements performed at a fixed frequency of 1 kHz and an applied voltage of 1 Vpp. Measurements were conducted in three configurations:

- Between fascicle wires, to characterize the intrinsic conductivity of the gel matrix.
- Between electrode needles, to assess electrode–simulator contact impedance.
- Between electrode needles and fascicle wires, to evaluate the interface impedance relevant to neural stimulation and signal acquisition.

**Figure 7:**
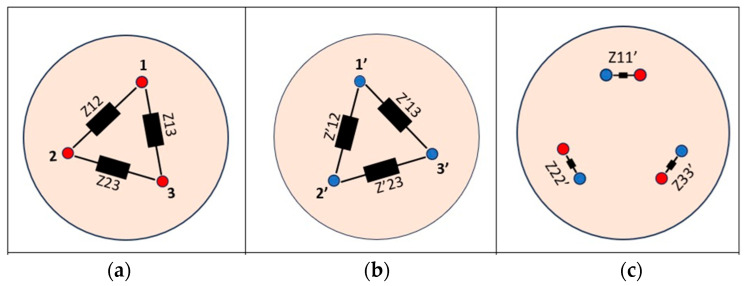
Experimental setup for impedance measurements between electrode needles. This configuration characterizes the electrode–simulator contact impedance under controlled conditions. (**a**) Between fascicle wires, to characterize the intrinsic conductivity of the gel matrix; (**b**) Between electrode needles, to assess electrode–simulator contact impedance; (**c**) Between electrode needles and fascicle wires, to evaluate the interface impedance relevant to neural stimulation and signal acquisition. Measured impedance values (~2.4–2.9 kΩ) closely match in vivo data, validating the simulator as a biomimetic testing platform for neural interfaces.

**Figure 8:**
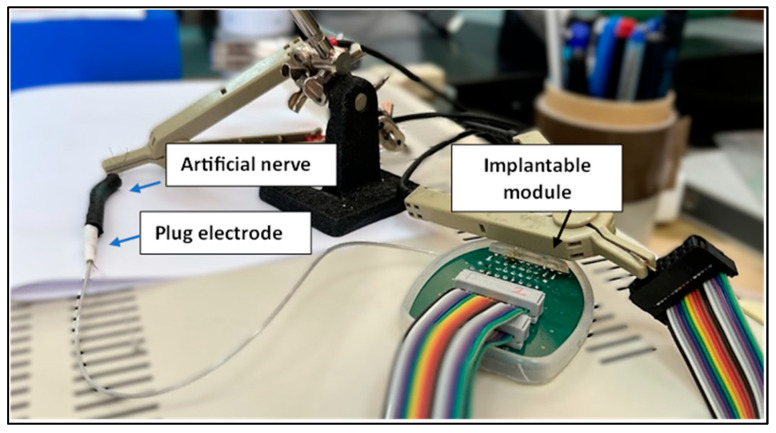
Experimental setup for impedance measurements between electrode needles and fascicle wires. This configuration evaluates signal transmission across the electrode–simulator interface. Measured impedance (<500 Ω) reflects direct conductive pathways within the hydrogel composite and confirms stable electrical coupling between embedded channels and electrode contacts.

Representative impedance measurement setups are shown in Figure 7 and Figure 8, and the corresponding quantitative results are summarized in Table 6.

The impedance between fascicle wires (Z_12_, Z_13_, Z_23_) was consistently measured at ~1.81–2.34 kΩ, indicating a stable conductive environment within the hydrogel matrix. The impedance between electrode needles (Z′_12_, Z′_13_, Z′_23_) was observed in the range of 2.4–2.9 kΩ, closely matching impedance values reported for peripheral nerve tissue in vivo (~2 kΩ at 1 kHz), as previously measured in porcine models (Table 6). Minor variations in measured impedance between electrode pairs were attributed to mechanical bending or positioning differences during needle insertion, highlighting the importance of precise alignment during testing.

The impedance measured between electrode needles and fascicle wires (Z′_11_, Z′_22_, Z′_33_) was significantly lower, remaining below 500 Ω. This behavior reflects the expected decrease in impedance across direct conductive paths within the hydrogel matrix, and the variations observed here are primarily related to the proximity and alignment accuracy of the electrode tips relative to the embedded wires.

By using the same fabrication protocol, the impedance of the obtained materials showed a very small variation of less than 10 Ω.

The results confirm that the developed artificial nerve simulator successfully reproduces the electrical characteristics of peripheral nerve tissue, particularly in terms of contact impedance and conductive pathway behavior. The close agreement with in vivo data demonstrates the simulator’s potential as a reliable platform for evaluating electrode–nerve interfaces under controlled laboratory conditions.

### 3.3. Relevance for Plug Electrode Evaluation and Surgical Training

The impedance characteristics observed in the artificial nerve simulator directly support its use as a platform for testing and optimizing plug-type electrodes—an alternative to conventional cuff electrodes for establishing bidirectional communication with peripheral nerves. Previous studies performed on porcine models using plug electrodes reported a contact impedance of approximately 2 kΩ at 1 kHz, and similar values were observed in the present ex vivo simulator. These findings confirm the simulator’s ability to replicate the electrical environment encountered in in vivo conditions and validate its suitability for pre-implantation testing of neural interfaces.

Additionally, the simulator’s structural stability and reproducible impedance characteristics make it a valuable tool for surgical training. The ability to repeatedly insert and test electrodes in a biomimetic environment enables surgeons to practice precise needle insertion techniques and evaluate insertion forces without the need for live animal tissue. The presence of discrete conductive channels further facilitates controlled experiments on electrode positioning, contact impedance, and stimulation efficiency.

To contextualize the performance of the developed simulator, impedance measurements were compared with data obtained previously from peripheral nerves of *Sus scrofa domesticus* using plug electrodes (Table 6). In those experiments, impedance values ranged from ~0.9 to 2.1 kΩ at frequencies between 500 Hz and 2 kHz, depending on the applied voltage. The simulator developed in this study exhibited impedance values of ~2.4–2.9 kΩ between electrodes and <500 Ω between electrodes and conductive channels, demonstrating close agreement with biological tissue in terms of magnitude and frequency-dependent behavior. These results confirm that the conductive hydrogel composite and embedded wire configuration replicate key electrical features of peripheral nerve tissue, enabling relevant and repeatable ex vivo testing.

### 3.4. Implications and Limitations

The results demonstrate that the artificial nerve simulator provides a reproducible, physiologically relevant, and ethically favorable platform for testing implantable neural interfaces. Its performance in terms of impedance closely mirrors that of peripheral nerves, validating its use for electrode design optimization, signal fidelity evaluation, and insertion studies. Furthermore, its simplicity and reproducibility make it a promising tool for standardizing ex vivo testing protocols prior to in vivo experimentation. However, certain limitations remain. The current simulator primarily replicates static electrical properties and does not capture dynamic physiological processes such as ion channel activity or action potential propagation. Additionally, while dehydration behavior was qualitatively observed, systematic time-resolved measurements of impedance and hydration state were not performed. Future work will aim to refine the material composition to better simulate time-dependent neural responses and integrate microfluidic control to mimic dynamic ionic environments, further increasing the simulator’s fidelity and applicability.

## 4. Integration into Neural Prosthetic Development Pipeline

One of the primary objectives of developing the artificial nerve simulator was to establish a functional bridge between early-stage materials and device design and pre-clinical validation of implantable neuroprosthetic systems. Its integration within the broader development workflow is exemplified by its role in the NerveRepack project, which focuses on creating a fully implantable bidirectional neural interface capable of connecting the peripheral nervous system with external prosthetic devices.

### 4.1. Context and Functional Role in the NerveRepack System

The NerveRepack system is designed to acquire neural signals from peripheral nerves, translate them into control commands for prosthetic actuators, and deliver sensory feedback from prosthetic sensors back to the user’s nervous system. Achieving this bidirectional communication requires high-performance neural electrodes that ensure stable signal acquisition and stimulation without damaging nerve tissue. While cuff electrodes are traditionally used, experimental studies have shown that plug-type electrodes —consisting of fine needle-like conductors inserted directly into the nerve fascicle—offer several advantages, including more selective interfacing, reduced stimulation thresholds, and improved signal fidelity.

Before surgical implantation, it is critical to validate the electrode–nerve interaction under conditions that closely replicate in vivo properties. The artificial nerve simulator developed in this work addresses this need by providing a bio-inspired testing platform where the impedance behavior, contact properties, and signal transmission characteristics of plug electrodes can be assessed ex vivo with high reproducibility. The system enables precise determination of contact impedance (~2 kΩ at 1 kHz), evaluation of electrode insertion behavior, and assessment of conductive coupling between electrodes and simulated fascicles.

### 4.2. Advantages for Iterative Design, Calibration, and Training

The integration of the nerve simulator into the neuroprosthetic development workflow provides several engineering and practical benefits:

- Iterative electrode optimization: Because the simulator reproduces relevant structural and electrical properties of peripheral nerves, multiple electrode geometries and materials can be evaluated and compared under controlled conditions before in vivo testing.
- Device calibration and benchmarking: The reproducible electrical properties of the simulator allow reliable calibration of acquisition electronics and amplifiers, as well as benchmarking of new electrode designs against existing solutions.
- Surgical training and insertion testing: Surgeons and engineers can practice electrode insertion procedures and evaluate insertion forces without relying on animal tissue, improving precision and reducing training variability.
- Reduced reliance on animal models: By enabling ex vivo testing of key device functions, the simulator significantly reduces the number of animal experiments required, aligning with ethical imperatives and regulatory trends in biomedical research.

### 4.3. Path Toward Standardization and Broader Use

The reproducibility, scalability, and physiologically relevant properties of the artificial nerve simulator suggest that it can play a broader role beyond the NerveRepack project. Its standardized geometry and stable impedance characteristics make it suitable as a benchmarking tool for different types of implantable electrodes, stimulation protocols, and signal acquisition systems. Moreover, the fabrication approach based on rGO–PANI–agarose composite hydrogels and embedded conductive channels is adaptable and can be tailored to mimic different nerve sizes and architectures, potentially extending the platform to various peripheral nerve targets. The integration of such bio-inspired simulators into the early stages of device development can accelerate translation from laboratory prototypes to clinical applications by enabling more rigorous pre-implantation testing, reducing variability, and providing a reproducible reference environment for performance evaluation. As neuroprosthetic technologies continue to evolve toward more complex, multi-channel, and adaptive systems, the availability of such platforms will be essential to support their safe, efficient, and ethical development.

## 5. Conclusions and Outlook

In this work, we have engineered and validated a bio-inspired artificial nerve simulator specifically designed for the ex vivo evaluation of implantable neural interfaces and plug electrodes. The simulator combines a conductive hydrogel composite of reduced graphene oxide (rGO), polyaniline (PANI), agarose, sucrose, and sodium chloride with embedded conductive channels that replicate the fascicular organization and ionic conduction properties of peripheral nerves. The resulting construct exhibits impedance values (~2.4–2.9 kΩ between electrodes and <500 Ω between electrodes and conductive channels at 1 kHz) that closely match those observed in vivo, demonstrating its suitability as a physiologically relevant test platform.

The artificial nerve simulator fulfills three critical engineering requirements for next-generation neuroprosthetic systems:

(1) Structural fidelity—the fascicle-mimicking geometry and hydrated hydrogel matrix reproduce the gross architecture of peripheral nerves;
(2) Electrical relevance—impedance behavior and conductive properties align closely with biological tissue;
(3) Experimental utility—the system enables reproducible electrode testing, insertion evaluation, and signal transmission assessment under controlled laboratory conditions.

Beyond serving as a platform for validating plug-type electrodes developed within the NerveRepack project, the simulator also offers substantial potential for iterative electrode optimization, surgical training, and device calibration, while reducing dependence on animal experimentation. Its reproducible properties and scalable fabrication approach make it a promising candidate for standardizing ex vivo testing protocols across a range of neuroprosthetic technologies.

Future work will focus on refining the composite formulation to better emulate dynamic neural responses, integrating microfluidic elements to simulate ionic flow and physiological variability, and expanding the simulator’s architecture to model a broader range of peripheral nerve types. By bridging materials engineering, bio-inspired design, and translational device development, this platform lays the foundation for safer, faster, and more efficient advancement of implantable neural interfaces toward clinical application.

## Figures and Tables

**Table 1:** Representative examples of artificial nerve simulators and phantoms reported in the literature. Materials, structural features, and main functional properties are listed to highlight the range of approaches used to reproduce nerve geometry and mechanical properties. Most reported models rely on inert polymers such as PDMS or silicone and lack the ionic conduction and fascicular organization achieved in this work.

Physical Characteristics	Human Nerve	Synthetic Analog (Example)	Reference
Diameter	2–6 mm	3 mm PDMS cylinder with internal channels	[13,15]
Fascicle structure	5–20 fascicles	10-channel molded silicone core	[16,17]
Surface elasticity	~10–100 kPa	Silicone or Ecoflex, matched moduli	[18,19]
Outer sheath texture	Smooth, vascularized	3D-printed sheath with nanogrooves	[20]

**Table 2:** Conductive materials and composites used in artificial nerve models. Examples include conductive polymers, carbon-based materials, and metallic fillers, with their typical conductivity values and fabrication compatibility summarized. These materials enable enhanced charge transport and electrode interfacing compared with purely mechanical phantoms.

Material	Conductivity (S/m)	Use in Nerve Models	Reference
PEDOT: PSS	~10–100	Coated channels for stimulation	[21,22]
Carbon nanotube pastes	1–10	Embedded electrodes	[23,24]
Silver-epoxy composites	>10^5^	Simulated axons or ground paths	[25]
Graphene ink	~10^2^	Printed signal pathways	[26]

**Table 3:** Fabrication techniques and structural features reported for nerve simulators. Methods such as soft lithography, extrusion-based 3D printing, and laser micromachining allow fabrication of cylindrical geometries and embedded conductive channels. The table highlights the range of achievable resolutions, channel designs, and mechanical properties.

Fabrication Technology	Resolution	Electrical Features Enabled	Reference
Soft lithography	~10 µm	Patterned microelectrodes	[27]
Extrusion-based 3D print	~100 µm	Channelized structure + conductive ink	[28]
Laser micromachining	~1 µm	Precision-cut waveguides	[29]

**Table 4:** Overview of electrical characterization protocols used for evaluating artificial nerve simulators. Techniques include impedance spectroscopy, pulse response testing, and multichannel signal mapping. Each method provides complementary information on conductivity, impedance, and signal propagation behavior, sup-porting design optimization of neural interface devices.

Test Type	Parameter Measured	Typical Setup	Reference
Signal mapping	Crosstalk between channels	Multichannel scope, 10–100 Hz sweeps	[30]
EIS (Impedance)	Z	@ 1 kHz	[33]
Pulse response	Delay, rise time	Square wave injection, oscilloscope	[34]

**Table 5:** Impedance and capacitance of peripheral nerve fascicles (Sus scrofa domesticus, ~50 kg) measured at different frequencies and applied voltages using plug-type electrodes. The contact impedance determined from these measurements was approximately 2 kΩ, which serves as a benchmark for the performance evaluation of the artificial nerve simulator.

Voltage	Frequency	Impedance	Capacitance	Voltage	Frequency	Impedance	Capacitance
Continuous nerve				Contiguous nerve			
1 V	500 Hz	2.1 kΩ	410 nF	2 V	500 Hz	1.5 kΩ	300 nF
1 V	1 kHz	0.88 kΩ	330 nF	2 V	1 kHz	1.5 kΩ	280 nF
1 V	1.5 kHz	1.2 kΩ	210 nF	2 V	1.5 kHz	2.1 kΩ	170 nF
1 V	2.0 kHz	0.9 kΩ	140 nF	2 V	2.0 kHz	2.1 kΩ	110 nF

**Table 6:** Impedance values measured on the artificial nerve simulator under different electrode configurations. Measurements were performed between fascicle wires (Z_12_, Z_13_, Z_23_), between electrode needles (Z′_12_, Z′_13_, Z′_23_), and between electrode needles and fascicle wires (Z′_11_, Z′_22_, Z′_33_). The impedance (~2.4–2.9 kΩ between electrodes) closely matches physiological values, validating the simulator as a bio-inspired platform for neural interface testing.

Simulator	Impedance Between Wires			Impedance Between Needles			Impedance Between Needles and Wires		
	Z_12_ (kΩ)	Z_13_ (kΩ)	Z_23_ (kΩ)	Z′_12_ (kΩ)	Z′_13_ (kΩ)	Z′_23_ (kΩ)	Z′_11_ (kΩ)	Z′_22_ (kΩ)	Z′_33_ (kΩ)
1	1.81	2.34	1.81	2.8	2.5	2.4	0.3	0.4	0.3
2	1.81	2.34	1.81	2.9	2.7	2.6	0.5	0.3	0.4

**Figure A1:**
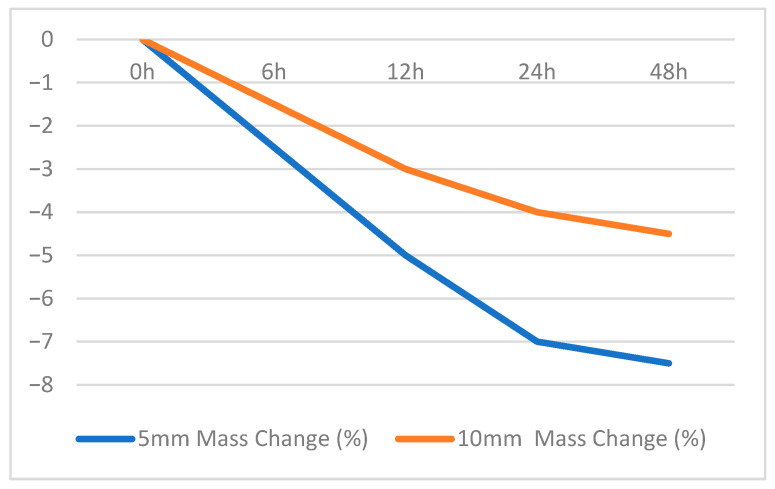
Dehydration/Desiccation Kinetics of Nerve Simulators.

**Figure A2:**
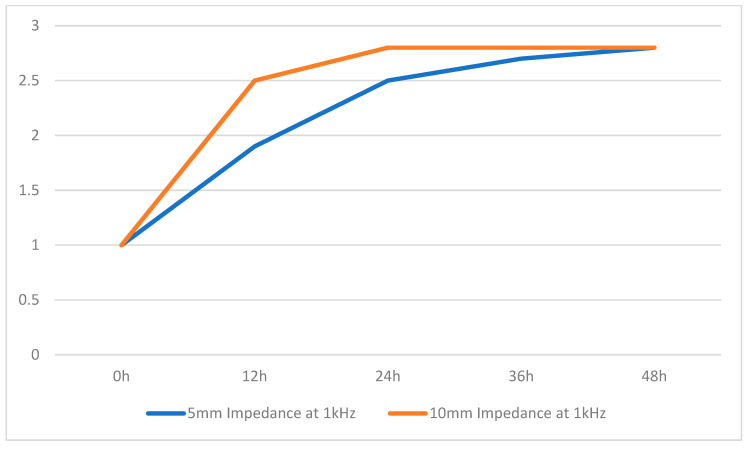
Impedance Stabilization Over Time (1 kHz).

## Data Availability

The data presented in this study are available on request from the corresponding author. The data are not publicly available due to the peculiarities of the software used to record them.

## Abbreviations

The following abbreviations are used in this manuscript:

3D	Three-Dimensional
cm	Centimeter
EIS	Electrochemical Impedance Spectroscopy
Hz	Hertz
IC	Integrated Circuit
kHz	Kilohertz
kΩ	Kiloohm
mm	Millimeter
µm	Micrometer
NaCl	Sodium Chloride
PANI	Polyaniline
PEDOT:PSS	Poly(3,4-ethylenedioxythiophene):poly(styrene sulfonate)
PDMS	Polydimethylsiloxane
rGO	Reduced Graphene Oxide
TPU	Thermoplastic Polyurethane
Vpp	Peak-to-Peak Voltage

## Appendix A

### Appendix A.1

The two samples exhibited two distinct and inversely correlated kinetic behaviors during the initial ex vivo stabilization period, as detailed in Figure A1 and Figure A2.

**Dehydration Kinetics**

The simulators were initially characterized for their mass stability under ambient laboratory conditions to quantify the rate of water loss and structural integrity (Figure 6).

As anticipated by fluid dynamics principles, the smaller 5 mm simulator exhibited a higher Surface Area-to-Volume (SA/V) ratio, resulting in a more rapid overall rate of water loss and a higher final percentage of mass change upon reaching its Equilibrium Water Content (EWC). This structure lost 7.5% of its initial mass within 48 h. Conversely, the 10 mm simulator, with its lower SA/V ratio, underwent slower bulk dehydration, stabilizing at a lower mass loss of 4.5% over the same period.

**Electrical Stabilization**

The electrical stability of the embedded conductive network was monitored concurrently by tracking the impedance magnitude at 1 kHz, a representative physiological frequency (Figure 7). Impedance is a critical performance parameter, as its stabilization signifies the end of the initial transient phase where the material and its ionic environment are adjusting to the external medium.

Contrary to the dehydration results, the larger 10 mm simulator achieved electrical stabilization significantly faster, reaching a stable impedance of in approximately 24 h. The smaller 5 mm simulator required nearly 48 h to reach the same stable impedance value.

**Discussion on Sample Evolution and Geometric Influence**

This counter-intuitive inverse correlation—where the structure with the slower bulk water loss (10 mm) stabilizes electrically faster—suggests that the rate-limiting step for electrical stabilization is not the bulk dehydration but rather the kinetics of the conductive network formation within the hydrogel matrix.

The temporal evolution of the test samples can be explained by two factors:

1. Ionic Condensation: The larger volume and mass of the 10 mm structure may result in greater internal compressive forces or localized thermal gradients during the final stages of curing and initial swelling/drying. These forces could lead to an earlier and more rapid coalescence of the hydrogel’s conductive components (e.g., polymer chains, charged groups) around the embedded wires.
2. Percolation Threshold: Electrical stabilization occurs when the concentration of the conductive phase (either the ionic medium or the conductive filler) reaches a percolation threshold relative to the electrode surface. It is hypothesized that in the larger 10 mm structure, the conditions necessary to reach this stable electrical state occur sooner, despite the overall slower rate of water extraction from the larger volume.

In conclusion, these results demonstrate a critical influence of geometric scaling on the functional performance of the bioelectronic interface. The 10 mm simulator exhibits a more desirable property for ex vivo testing—a faster time to functional electrical stability—despite its bulk physical properties (SA/V ratio) suggesting otherwise. These findings have practical implications for designing simulators intended for immediate or prolonged testing protocols.

The internal conductive components for both the 5 mm and 10 mm nerve simulators were fabricated using bare 316 L Stainless Steel wires with a diameter of approximately 100 µm. This material was chosen for its established biocompatibility and durability, aligning with common standards for medical-grade research components. A total of three wires were arranged symmetrically within the cylindrical mold/bioprinting path to emulate the discrete fascicular conduction pathways of a peripheral nerve. During the hydrogel casting and curing process, the wires were held straight using a custom-designed acrylic jig that applied a minimal, non-quantified pre-tension sufficient only to maintain linear alignment and prevent structural deformation. Crucially, the wires were deliberately left un-insulated to ensure that electrical current propagation occurred equally through the conductive metal core and the surrounding ionic hydrogel volume. This design choice facilitates the measurement of bulk tissue impedance and its stabilization dynamics, validating the simulator as a representative volume conductor model for ex vivo testing.

The diameter of the artificial nerve significantly influences its electrical behavior, mainly through its effects on dehydration kinetics, ionic conductivity stabilization, and impedance characteristics. Simulators with larger diameters (e.g., 10 mm) tend to reach resistance stabilization faster than smaller ones (e.g., 5 mm) due to the increased surface area that accelerates dehydration processes. This dehydration reduces the water content in the hydrogel, thereby diminishing the initial ionic mobility but leading to a more stable conductive network over time. In addition, the increased diameter increases the total cross-sectional area for ionic current flow, which can reduce impedance and facilitate more efficient signal transmission. However, the larger volume may prolong the time required to reach equilibrium conditions related to ion distribution and moisture content. Consequently, size optimization should balance rapid stabilization and efficient conduction properties, mimicking the physiological characteristics of peripheral nerves.

The tensioned wires incorporated into the artificial nerve simulator serve to mimic the structure and function of biological nerve bundles. Specifically, they are positioned along the hydrogel-based model to simulate nerve bundles and conduct currents similar to those in biological nerves. These wires help reproduce the ionic conduction pathways in nerve tissue, allowing for realistic evaluation of electrical properties and signal transmission within the artificial construct. Tensioning them ensures proper alignment and structural stability, facilitating consistent experimental conditions for electrical characterization and ensuring that the simulator accurately reflects the behavior of natural peripheral nerves.

### Appendix A.2

**Table A1:** Representative artificial nerve models and test platforms reported in the literature, compared by materials, fabrication, geometry, electrical properties, and main application.

Study (DOI)	Materials/Composite	Geometry & Dimensions	Fabrication Method	Electrical Properties	Primary Application/Notes
Koutsouras et al., 2019—10.1002/adhm.201901215	PEDOT:PSS hydrogel + PDMS	3 mm diameter, 5 channels	Molded elastomer, gel filling	Conductivity: 1–10 S/m; Impedance mapping	Selective stimulation and channel impedance profiling
Lienemann et al., 2021—10.1088/1741-2552/abfebb	Gold nanowires + epoxy	4 mm diameter, 8 cm length	Direct-write 3D printing	Square-wave propagation, impedance spectra	Signal propagation and impedance evaluation
Convertino et al., 2023—10.3389/fbioe.2023.1306184	Graphene–polymer composite	2.5 mm diameter, 6 channels	Inkjet printing, layered concentric structures	Phase delay, action potential simulation	Signal delay modeling and demyelination studies
Rosati et al., 2007—10.1109/TNSRE.2007.908426	Silicone + carbon ink	5 mm diameter, 3 segments	Layered lithography	Segmental impedance mapping	Nerve regeneration scaffold testing
Vlasceanu et al., 2019—10.1016/j.compositesb.2019.106977	Graphene ink + TPU	3 mm diameter, 10 cm length	Multimaterial extrusion printing	Crosstalk and voltage attenuation	Mechanical–electrical coupling studies
Papadimitriou et al., 2020—10.1016/j.mtbio.2020.100043	Nano-textured PDMS	3.5 mm diameter, single core	Soft lithography + plasma etching	Voltage threshold modulation	Surface topology effects on stimulation
Niu et al., 2021—10.1016/j.bios.2021.113506	Acrylic + gold leaf	2 mm diameter, 4 fascicles	Laser micromachining	Signal fidelity and conduction velocity	High-resolution signal conduction modeling
Chang et al., 2023—10.1016/j.electacta.2023.142741	Silver epoxy + PDMS	6 mm diameter, variable length	Embedded conductor casting	EIS across multiple frequency ranges	Benchmarking commercial cuff electrodes
Ionescu et al., 2024—10.3390/bios14010031	Plug-type electrodes in Sus scrofa model	Peripheral nerve interfacing	Implantation and in vivo testing	Impedance ≈ 2 kΩ @ 1 kHz	Benchmark in vivo performance reference
